# Causal role of MiRNAs in chronic rhinosinusitis: mendelian randomization and validation study

**DOI:** 10.1186/s13223-025-00957-4

**Published:** 2025-04-17

**Authors:** Lei Shi, Yi-ran Zhao, Zhi-xuan Ma, Fu Shu

**Affiliations:** 1 Department of Otorhinolaryngology, The Affiliated Hospital of Liaoning University of Traditional Chinese Medicine, No. 33 Beiling Street, Huanggu District, Shenyang, Liaoning 110847 People’s Republic of China; 2 School of Chinese Medicine, Hong Kong Special Administrative Region, Hong Kong Baptist University, Hong Kong, People’s Republic of China; 3 College of Traditional Chinese Medicine, Chongqing Medical University, No. 1 YiXueYuan Road, Yuzhong District, Chongqing, 400000 People’s Republic of China

**Keywords:** Chronic rhinosinusitis, Mendelian randomization, MiRNA, MAPK pathway, PI3K-AKT pathway

## Abstract

**Background:**

Despite significant advances in understanding the epigenetic landscape of chronic rhinosinusitis (CRS), the specific microRNAs (miRNAs) with a causal role in CRS pathogenesis remain unclear.

**Objective:**

This study aims to identify miRNAs that causally contribute to CRS and to elucidate their clinical relevance and underlying molecular mechanisms.

**Methods:**

We employed Mendelian randomization (MR) analysis, leveraging mirQTLs as exposure variables and two independent CRS datasets as outcomes, to identify miRNAs causally linked to CRS. Robustness of the findings was ensured through multiple sensitivity analyses. The expression levels of identified CRS-associated miRNAs were validated using qRT-PCR, and their diagnostic potential was assessed through ROC curve analysis. Target genes and potential pathways regulated by the causal miRNAs were predicted via MiRNet and enrichment analyses, followed by experimental validation using western blotting and immunohistochemistry.

**Results:**

MiR-130a-3p and miR-196b-5p were significantly associated with an increased risk of CRS, while miR-339-3p was associated with a decreased risk. These associations were confirmed by qRT-PCR, and no evidence of pleiotropy or heterogeneity was observed. ROC analysis revealed diagnostic potential for these miRNAs in CRS. Enrichment and experimental analyses suggested that the MAPK and PI3K-AKT pathways are predominantly activated by the target genes of the positively and negatively associated miRNAs, respectively.

**Conclusions:**

MiR-130a-3p and miR-196b-5p are positively associated with CRS risk, whereas miR-339-3p is protective. These miRNAs represent promising diagnostic biomarkers and therapeutic targets for CRS. The MAPK and PI3K-AKT pathways likely mediate the effects of these causal miRNAs, offering further insight into the molecular mechanisms underlying CRS.

**Supplementary Information:**

The online version contains supplementary material available at 10.1186/s13223-025-00957-4.

## Background

Chronic rhinosinusitis (CRS) is a complex upper respiratory tract disorder characterized by persistent inflammation of the sinus mucosa. It is commonly classified into two clinical phenotypes: CRS with nasal polyps (CRSwNP) and CRS without nasal polyps (CRSsNP) [1–3]. Despite the widespread use of established treatments, including intranasal corticosteroids and endoscopic sinus surgery [4], many patients continue to experience persistent or recurrent symptoms even after receiving standard care [5]. The precise molecular mechanisms underlying CRS remain incompletely understood, significantly hampering the development of new therapeutic strategies [6]. Therefore, a deeper understanding of the key molecules involved in CRS pathogenesis and the identification of potential biomarkers is crucial for advancing novel treatment approaches.

MicroRNAs (miRNAs) are small non-coding RNAs that regulate gene expression at the post-transcriptional level by targeting messenger RNA (mRNA) [7, 8]. Recent research has increasingly demonstrated that dysregulated miRNAs play a significant role in the pathogenesis of CRS, influencing both molecular and phenotypic aspects of the disease. miRNAs are implicated in the activation of pro-inflammatory signaling pathways, immune cell regulation, and tissue remodeling processes in CRS [9–13]. Additionally, miRNAs possess unique expression profiles, critical regulatory functions, high stability, and target specificity in biological samples, making them promising candidates as diagnostic and therapeutic biomarkers for CRS [14, 15]. However, despite notable advancements in miRNA research related to CRS in recent years, the field remains in its early stages, with substantial gaps in our understanding of miRNAs with causal effects on CRS [16]. Most existing studies have identified correlations between miRNAs and CRS but have not investigated causal relationships. Unlike miRNA-based therapies for diseases such as stroke, which are in clinical development, miRNA research in CRS has yet to reach the stage of clinical trials and application [16, 17].

Mendelian randomization (MR) is a powerful method for inferring causal relationships between exposures and outcomes by using genetic variants as instrumental variables (IVs) [18]. Since genetic variants are randomly assigned at conception, MR can effectively minimize confounding bias. Additionally, because these variants are determined before disease onset, MR can prevent reverse causality, addressing limitations inherent in observational studies [19]. Previous MR studies have assessed the causal relationships between miRNAs and various diseases, including Parkinson’s disease [20], amyotrophic lateral sclerosis [21], schizophrenia [22], and COVID-19 [23]. However, no MR studies have yet evaluated the causal relationship between miRNAs and CRS, leaving the mechanisms by which miRNAs influence CRS risk largely unexplored.

**Fig. 1:**
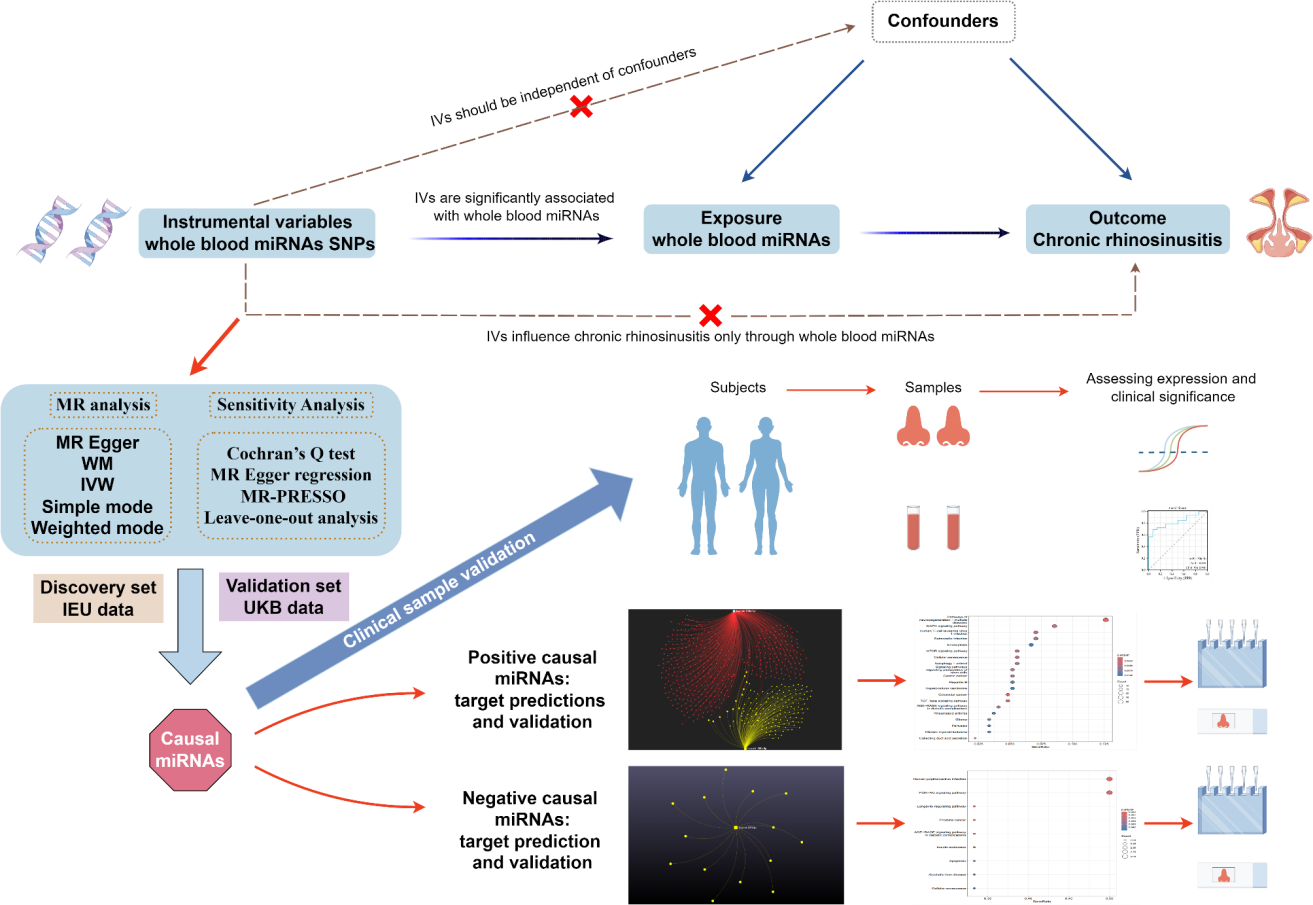
Research workflow diagram (By Figdraw). IVs: instrumental variables; microRNAs: miRNAs; WM: Weighted Median; IVW: inverse-variance weighted; IEU: Integrative Epidemiology Unit; UKB: UK Biobank

In this study, we aimed to assess the potential of miRNAs as predictors and therapeutic targets for CRS by employing a two-sample MR approach. We utilized summary data from genome-wide association studies (GWAS), with miRNA expression quantitative trait loci (mirQTLs) as exposure variables, and two independent CRS datasets as discovery and validation outcomes. Our goal was to identify miRNAs with causal effects on CRS and to elucidate their clinical significance and downstream effects through a combination of clinical sample analysis, bioinformatics, and molecular biology methods (Fig. 1).

## Methods

### MR analysis

MR analysis was conducted using the “TwoSampleMR” R package to identify miRNAs with causal effects on CRS [24]. For exposure data, we utilized mirQTL study data from Huan et al., which included 5,239 participants from the Framingham Heart Study (FHS) [25]. To minimize pleiotropy, we selected cis-mirQTLs and excluded single nucleotide polymorphisms (SNPs) located in synonymous or missense coding regions [23]. The CRS dataset with the widest genotype coverage (ID: ebi-a-GCST90018823) from the Integrative Epidemiology Unit Open GWAS project was used as the discovery set, while CRS data from the UK Biobank (UKB), diagnosed based on ICD-10 (ID: ukb-d-J32), was used for validation. Following established protocols [21], summary association statistics for SNPs independently associated with miRNAs were compiled using a clumping window of 10 kb and an r² cutoff of 0.5, with a significance threshold set at *P* < 6.6 × 10^− 5^. These statistics served as IVs to explore potential causal relationships in the outcome GWAS. The inverse-variance weighted (IVW) method, recognized for its power in detecting causal effects, was used as the primary analysis method [26]. Sensitivity analyses were conducted using MR Egger, Weighted Median, and MR-PRESSO methods to confirm the robustness of the IVW results [27]. Pleiotropy was assessed using MR-Egger regression and MR-PRESSO [28], while heterogeneity was evaluated with Cochran’s Q test [29]. The leave-one-out method was applied to determine if results were driven by a single SNP [30], and the F-statistic was calculated to assess the influence of weak instruments [31]. To account for multiple testing, a Bonferroni-corrected significance level was applied in the discovery set (*P* <.00067). In the validation set, a P-value < 0.05 was considered significant.

### Clinical sample collection

Nasal mucosa and blood samples were collected from 14 patients undergoing septoplasty and 32 patients diagnosed with CRSwNP according to the EPOS guidelines [32]. Nasal mucosa samples from septoplasty patients were obtained from the uncinate process or middle turbinate, while samples from CRSwNP patients were collected from nasal polyps. Blood samples were drawn using BD PAXgene Blood RNA Tubes (Becton, Dickinson and Company, USA) from 2.5 ml of fasting morning blood. Exclusion criteria included a history of fungal sinusitis, cystic fibrosis, recent use of antibiotics, immunomodulators, or corticosteroids within the past month, as well as pregnancy or lactation. Demographic and clinical data of the participants are listed in Supplementary Table S1.

### Quantitative Real-Time PCR (qRT-PCR)

Total RNA was extracted from clinical tissue samples using Trizol reagent according to the manufacturer’s instructions. Blood miRNAs were purified using the PAXgene Blood miRNA Kit. Reverse transcription of total RNA was performed using the miRNA First Strand cDNA Synthesis Kit, followed by qRT-PCR using the SYBR Green qPCR Kit. U6 was used as an internal control, and relative gene expression levels were calculated using the 2-^ΔΔCt^ method. Primer sequences are provided in Supplementary Table S2.

### Clinical value analysis

The diagnostic performance of the causal miRNAs in distinguishing healthy individuals from CRS patients was assessed using ROC curve analysis with GraphPad Prism 9. The area under the curve (AUC) was calculated to determine diagnostic accuracy.

### Target gene prediction, functional annotation analysis, and experimental validation

To explore the functional implications of the causal miRNAs identified through MR, target genes were predicted using miRNet 2.0, including only experimentally validated targets from the miRTarBase V8.0 database [21, 33]. GO and KEGG enrichment analyses for the target genes of both positive and negative causal miRNAs were conducted using the “clusterProfiler” R package, with significant GO terms and KEGG pathways identified at a corrected P value < 0.05. Key pathways identified from the KEGG analysis were further validated through Western Blotting (WB) and Immunohistochemistry (IHC).

### WB

Total protein was extracted from nasal mucosa and nasal polyp tissues using RIPA Lysis Buffer and quantified with the BCA Protein Assay Kit. Protein samples were normalized and denatured by heating at 95 °C for 5 min. Proteins were separated by sodium dodecyl sulfate-polyacrylamide gel electrophoresis (SDS-PAGE) and transferred onto polyvinylidene fluoride (PVDF) membranes. Membranes were blocked with 10% non-fat milk for 2 h at room temperature, incubated with primary antibodies overnight at 4 °C, washed with TBST, and then incubated with secondary antibodies for 2 h at room temperature. Protein bands were visualized using the Ultrasensitive ECL Detection Kit and quantified with Image J software. GAPDH was used as an internal control. Relative protein expression levels were calculated by comparing the gray values of the target protein bands to those of the internal control bands. For phosphorylated proteins, relative phosphorylation levels were calculated by comparing the gray values of phosphorylated protein bands to those of the total protein bands. Antibody details are provided in Supplementary Table S3.

### IHC

Paraffin-embedded human nasal mucosa and nasal polyp tissues were sectioned into 4 μm thick slices. Antigen retrieval was performed using 0.01 M citrate buffer (pH 6.0), followed by blocking of endogenous peroxidase activity with 3% hydrogen peroxide (H₂O₂) solution. After blocking with 5% goat serum, primary antibodies were applied to the sections and incubated overnight at 4 °C. The following day, biotinylated secondary antibodies were applied and incubated at room temperature for 1 h. Sections were stained using a DAB staining kit, lightly counterstained with hematoxylin, and mounted with neutral gum. Staining intensity was evaluated to determine protein expression levels.

### Statistical analysis

All data were analyzed using GraphPad Prism 9. Continuous variables with a normal distribution were expressed as mean ± standard deviation (SD), while non-normally distributed continuous variables were expressed as median (interquartile range). Comparisons between two groups for normally distributed data were conducted using an independent samples t-test, while the Mann-Whitney U test was used for non-normally distributed data. Categorical variables were described as frequencies and percentages, with differences between groups evaluated using the Chi-square test or Fisher’s exact test. Statistical significance was set at *P* <.05.

## Results

### Characteristics of IVs in MR

We initially identified 9,612 potential SNPs from the FHS data as IVs. After excluding 95 SNPs located in synonymous or missense coding regions, and 17 SNPs associated with miR-213 due to the absence of a valid miRNA ID, 9,500 SNPs significantly associated with human miRNA expression were retained for further analysis. In the IVW analysis of the discovery cohort, detailed information on all SNPs used in the positive MR analyses is provided in Supplementary Table S4. These findings were then validated in the validation cohort, with additional details listed in Supplementary Table S5. Notably, the F-statistics for all SNPs exceeded 10 in both the discovery and validation cohorts, indicating that the IVs employed in this study were not affected by weak instrument bias.

### MR results

**Fig. 2:**
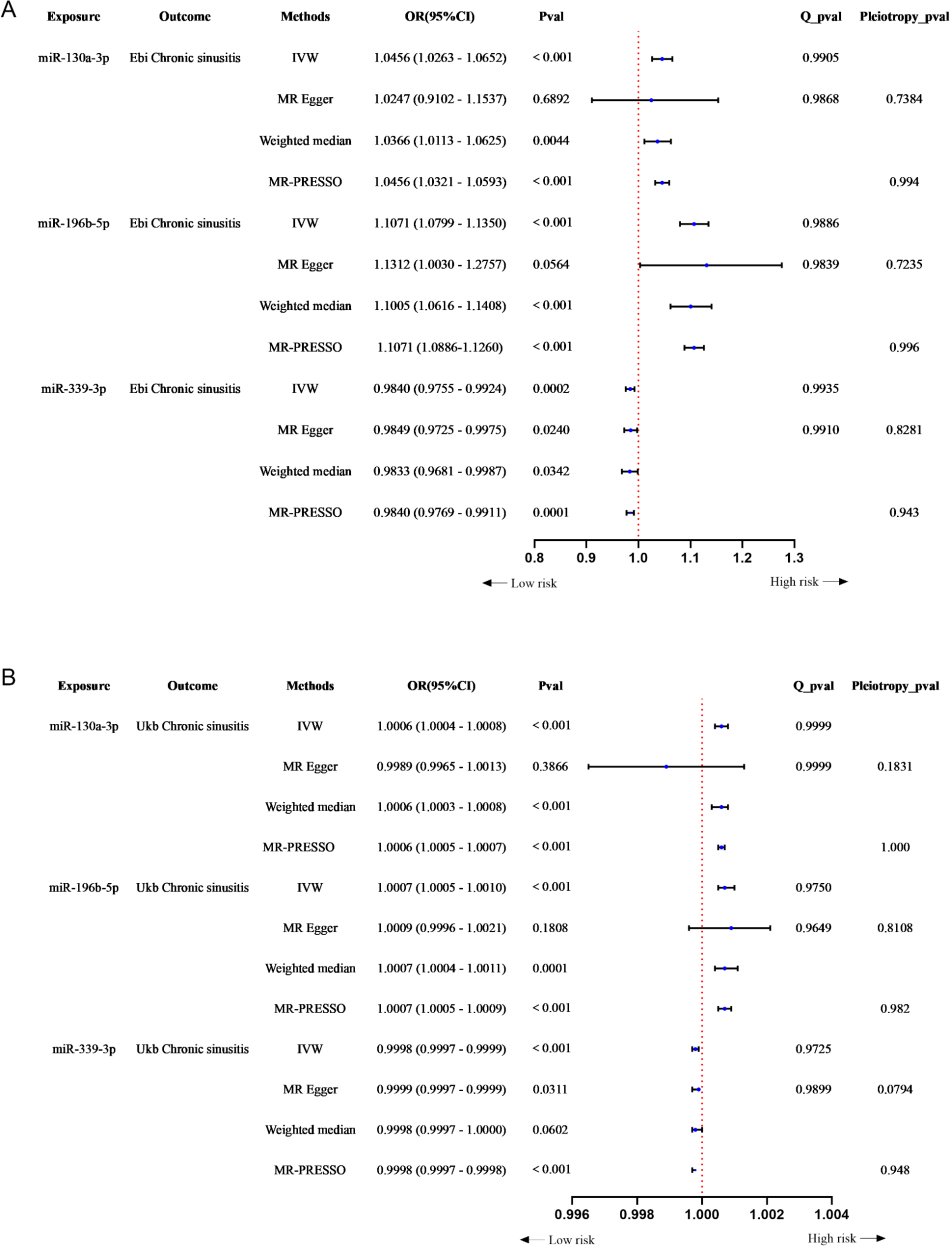
Protective and harmful effects of miRNAs on CRS risk. (**A**) discovery cohort; (**B**) validation cohort. OR: odds ratios; CI: confidence intervals; IVW: inverse-variance weighted

**Fig. 3:**
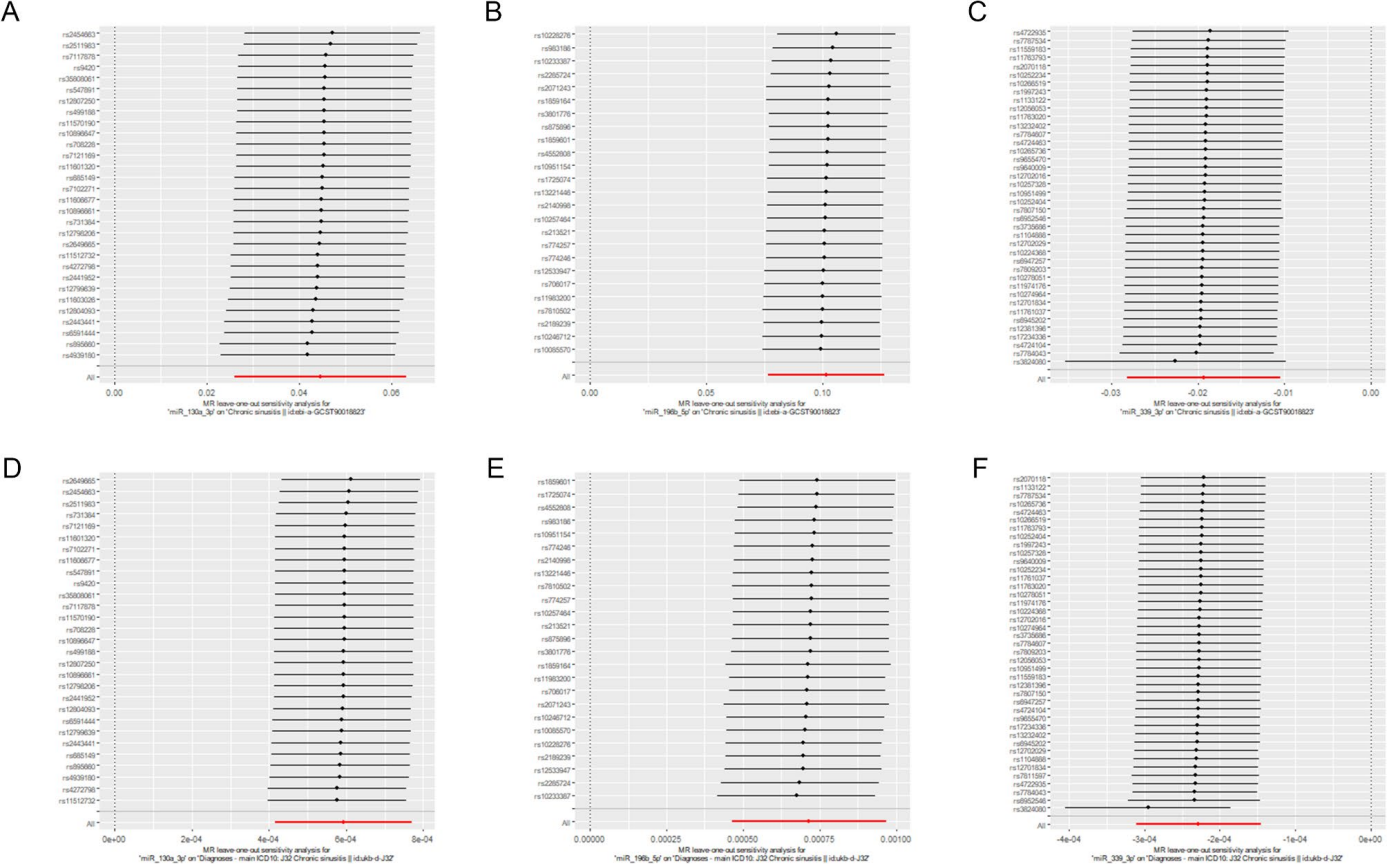
Leave-one-out analysis of miRNAs on CRS risk. (**A**-**C**) discovery cohort; (**D**-**F**) validation cohort

Figure 2A and S1A-S1C illustrate significant positive causal relationships between miR-130a-3p and miR-196b-5p and the risk of CRS, while miR-339-3p exhibited a significant negative causal relationship with CRS risk in the Ebi discovery cohort. The analysis of the UKB validation dataset, as shown in Fig. 2B and S1D-S1F, demonstrated consistent causal effects. Additionally, no evidence of horizontal pleiotropy or heterogeneity was observed in either the discovery or validation cohorts (Fig. 2A and B). The leave-one-out analyses indicated that the causal effects observed in the MR were not driven by any single SNP (Fig. 3A and F). These findings robustly support the stability and reliability of the MR results.

### Expression validation and clinical significance of causal MiRNAs

**Fig. 4:**
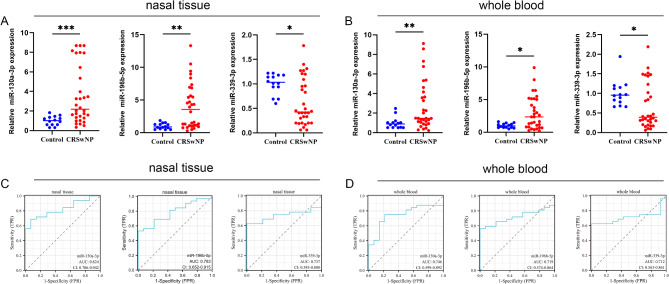
qPCR and ROC analysis of miRNAs causally associated with CRS. (**A**) qPCR expression of miRNAs in nasal tissues; (**B**) qPCR expression of miRNAs in blood samples; (**C**) ROC curve based on nasal qPCR expression; (**D**) ROC curve based on blood qPCR expression. *P <.05, **P <.01, ***P <.001

qRT-PCR expression analysis revealed that miR-130a-3p and miR-196b-5p were significantly upregulated in both blood and nasal tissue samples compared to controls, while miR-339-3p was significantly downregulated in both blood and nasal tissue samples (Fig. 4A and B, respectively). ROC analysis demonstrated that the AUC for miR-130a-3p, miR-196b-5p, and miR-339-3p in nasal tissue were 0.824, 0.783, and 0.737, respectively, and in whole blood were 0.746, 0.719, and 0.712, respectively (Fig. 4C and D). These results indicate that the causal miRNAs possess substantial accuracy in clinical diagnosis.

### Functional prediction and experimental validation of causal MiRNAs

**Fig. 5:**
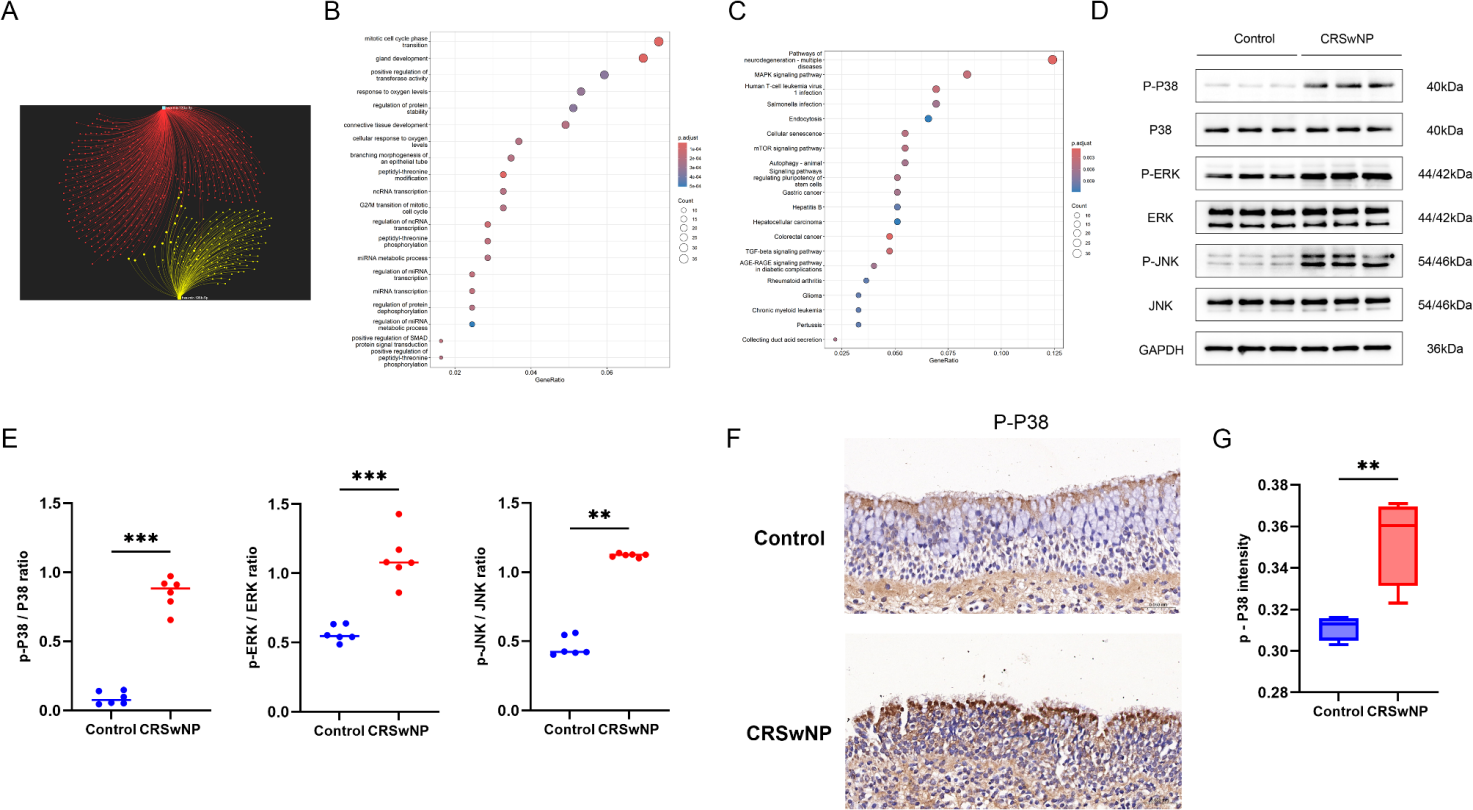
Target Gene Predictions, Enrichment Analyses, and Experimental Validations of miRNAs Positively Associated with CRS. (**A**) Predicted target genes of miRNAs with a positive causal effect on CRS; (**B**-**C**) GO enrichment analysis (Biological Process) and KEGG pathway enrichment analysis of the predicted target genes; (**D**-**E**) Western blot validation and statistical analysis of the MAPK signaling pathway activated by the target genes; (**F**-**G**) Immunohistochemistry validation and statistical analysis of the MAPK signaling pathway activated by the target genes

To explore the mechanisms by which the positive causal miRNAs, miR-130a-3p and miR-196b-5p, may increase CRS risk, we predicted their target genes. A total of 535 genes were predicted as targets for these two miRNAs (Fig. 5A). These target genes were significantly enriched in biological processes and pathways associated with CRS, such as autophagy, MAPK, and TGF-β signaling pathways (Fig. 5B and C). WB and IHC validation confirmed that the main branches of the MAPK pathway (P38, ERK, and JNK) were significantly activated in CRS patients compared to controls (Fig. 5D and G).

**Fig. 6:**
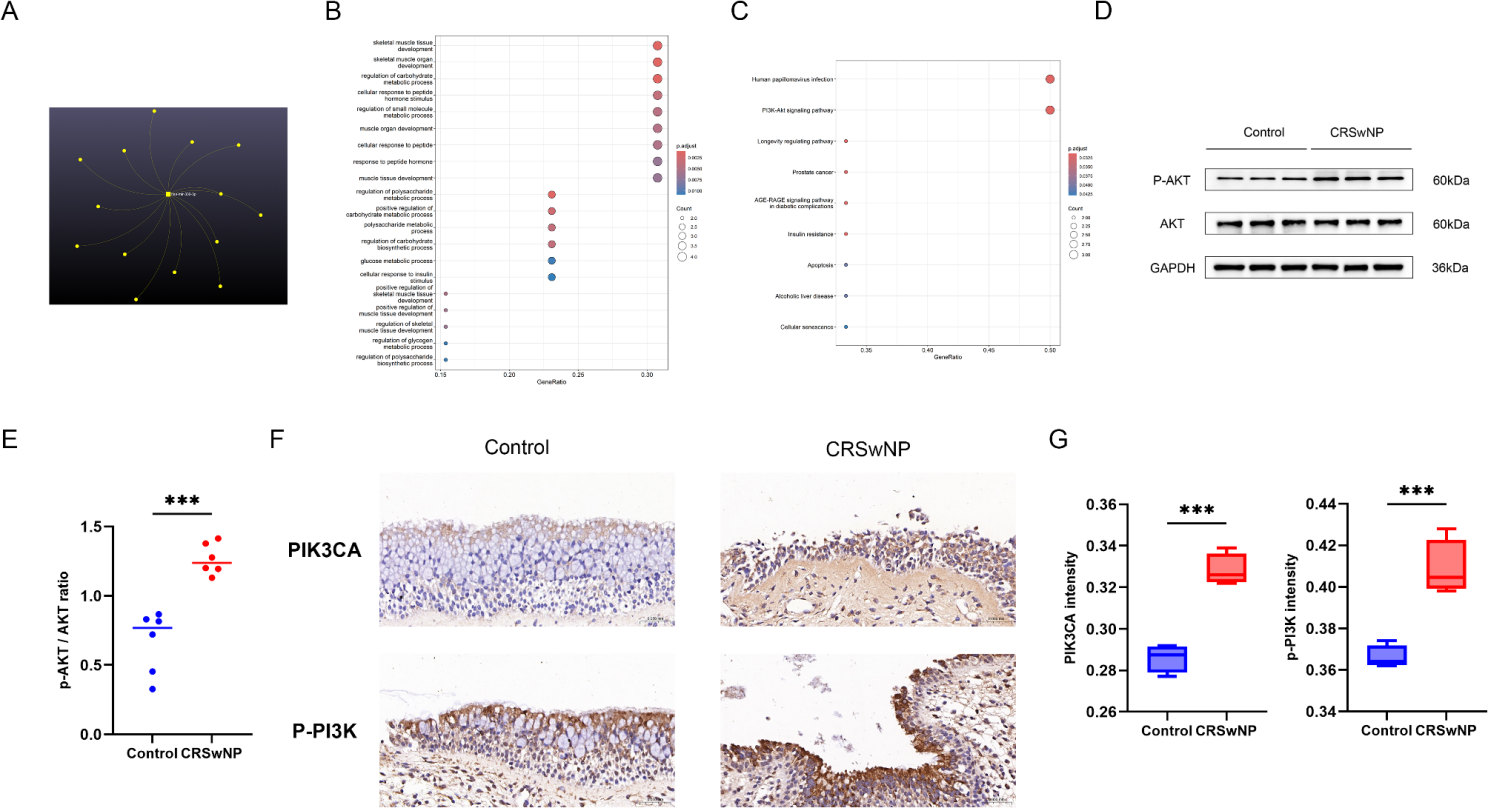
Target Gene Predictions, Enrichment Analyses, and Experimental Validations of miRNAs Negatively Associated with CRS. (**A**) Predicted target genes of miRNAs with a negative causal effect on CRS; (**B**-**C**) GO enrichment analysis (Biological Process) and KEGG pathway enrichment analysis of the predicted target genes; (**D**-**E**) Western blot validation and statistical analysis of the PI3K-AKT signaling pathway activated by the target genes; (**F**-**G**) Immunohistochemistry validation and statistical analysis of the PI3K-AKT signaling pathway activated by the target genes

Similarly, to investigate the protective mechanism of the negative causal miRNA, miR-339-3p, we predicted its target genes. A total of 14 genes were predicted as targets for miR-339-3p (Fig. 6A). These target genes were significantly enriched in biological processes and pathways related to CRS, such as apoptosis, the PI3K-Akt signaling pathway, and carbohydrate metabolism (Fig. 6B and C). WB and IHC validation demonstrated that the PI3K-Akt signaling pathway was significantly activated in CRS patients compared to controls (Fig. 6D and G).

## Discussion

In this study, we employed MR analysis, combined with bioinformatic predictions and experimental validations, to identify miR-339-3p as having a protective effect against CRS, while miR-130a-3p and miR-196b-5p were identified as causal molecules associated with an increased risk of CRS. These findings were consistently observed across both the discovery and validation cohorts, with expression validation further corroborating these results. Additionally, the target genes of both the positive and negative causal miRNAs were significantly enriched in biological processes related to CRS, and experimental validation confirmed the activation of these signaling pathways. These results suggest a potentially crucial role for these miRNAs in the pathogenesis of CRS.

Despite significant advancements in medical and surgical treatments, the complexity and heterogeneity of CRS pose major challenges in its management, contributing to its substantial public health burden [34]. Given the potential of miRNAs as effective biomarkers and therapeutic targets for CRS, identifying those with causal associations with the disease is essential for advancing new biomarkers and therapeutic strategies [35].

To date, no studies have explored the relationship between miR-339-3p and CRS, leaving its role in the disease unclear. However, our MR analysis and differential expression analysis of clinical samples strongly indicate that miR-339-3p may have a negative causal effect on CRS, correlating with a reduced risk of the disease. Previous research has established that the pathogenesis of CRS is closely related to apoptosis and the PI3K-AKT signaling pathway [36–38]. miR-339-3p is strongly associated with apoptosis [39], and our target gene enrichment analysis revealed significant involvement of apoptosis and the PI3K-AKT signaling pathway. Experimental validation demonstrated significant activation of the PI3K-AKT signaling pathway in CRS patients, consistent with previous findings that activation of this pathway promotes apoptosis in nasal epithelial cells [40]. These findings suggest that miR-339-3p may influence apoptosis in CRS through the PI3K-AKT signaling pathway. Additionally, miR-339-3p is significantly downregulated in nasopharyngeal carcinoma tissues, and its upregulation can inhibit the growth and epithelial-mesenchymal transition process of nasopharyngeal carcinoma cells [41]. Given the significant correlation between CRS and nasopharyngeal carcinoma reported in recent meta-analyses, these findings further support the potential role of miR-339-3p in CRS. The ROC curve results for miR-339-3p also suggest its clinical utility in diagnosing CRS, making it a promising therapeutic target and biomarker.

Our MR analysis also revealed a positive causal relationship between elevated levels of miR-130a-3p and miR-196b-5p and the risk of CRS. Differential expression analysis in both blood and nasal tissue further demonstrated that miR-130a-3p and miR-196b-5p were significantly upregulated in CRS patients compared to controls. Although the differential expression samples were from an Asian population, the consistent expression trends with MR analysis further support the causal relationship between these miRNAs and CRS. The ROC curve results indicate that miR-130a-3p and miR-196b-5p can effectively distinguish CRS patients from healthy controls, underscoring their clinical diagnostic potential. This is consistent with previous research showing miR-130a-3p’s diagnostic and prognostic value in acute ischemic stroke and miR-196b-5p’s role in early renal cell carcinoma [42, 43]. Although current research on these miRNAs primarily focuses on tumors, miRNAs regulate gene expression by binding to the 3’UTR of target genes, influencing multiple biological processes and playing significant roles in disease pathophysiology. Given that autophagy defects have been linked to chronic mucosal inflammation in CRS [44], and the involvement of the MAPK pathway in CRS pathogenesis, we hypothesize that miR-130a-3p and miR-196b-5p, which are positively associated with CRS, may influence autophagy through the activation of pathways such as MAPK, thereby contributing to CRS pathogenesis.

This study presents several innovative aspects compared to existing literature. First, we utilized MR analysis, which can simulate randomized controlled trials and effectively avoid reverse causation and residual confounding, providing robust evidence for the causal associations between miRNAs and CRS. Second, this is the first study in the field of CRS to systematically explore the role of miRNAs in CRS pathogenesis from a causal perspective, identifying three miRNAs with causal associations with CRS. By combining bioinformatics and experimental validation, we elucidated the downstream mechanisms and clinical significance of these miRNAs, offering new insights into CRS pathogenesis and the development of novel therapeutic strategies and biomarkers. Lastly, by employing both discovery and validation cohorts and conducting comprehensive sensitivity analyses, we found no significant heterogeneity or pleiotropy that could potentially bias the results, thereby supporting the stability and reliability of our findings.

However, several limitations should be considered when interpreting our results. First, the limited number of miRNAs in the miRNA eQTL data precludes a comprehensive assessment of the causal relationships between all identified miRNAs and CRS. Second, although MR analysis is a powerful tool for causal inference, the specific roles of the miRNAs with causal associations in CRS require further validation through experimental methods, such as gene knockout mouse models or the use of adeno-associated viruses.

## Conclusions

In summary, we identified three miRNAs with causal associations with CRS, positioning them as potential biomarkers for the disease. The positive causal miRNAs may increase CRS risk by regulating biological processes such as autophagy and modulating signaling pathways like MAPK. Conversely, the negative causal miRNA may offer protection against CRS by influencing apoptosis and the PI3K-AKT signaling pathway. These findings not only deepen our understanding of CRS pathogenesis but also suggest new avenues for the diagnosis and treatment of CRS. Furthermore, this study introduces a novel perspective and methodology for investigating the etiology of complex diseases.

## Electronic supplementary material

Below is the link to the electronic supplementary material.

Supplementary Material 1

## Data Availability

The mirQTLs data are available for direct download from the supplementary materials of the published article (https://www.ncbi.nlm.nih.gov/pmc/articles/PMC4369777/). GWAS data on chronic sinusitis are publicly accessible and can be retrieved from the IEU website(https://gwas.mrcieu.ac.uk/) by searching for GWAS ID: ebi-a-GCST90018823 and ID: ukb-d-J32.

## Abbreviations

CRS: Chronic rhinosinusitis
CRSwNP: CRS with nasal polyps
CRSsNP: CRS without nasal polyps
miRNAs: MicroRNAs
mRNA: Messenger RNA
MR: Mendelian randomization
IVs: Instrumental variables
GWAS: Genome-wide association studies
mirQTLs: Mirna expression quantitative trait loci
FHS: Framingham heart study
SNP: Single nucleotide polymorphism
UKB: UK Biobank
IVW: Inverse-variance weighted
qRT-PCR: Quantitative real-time PCR
AUC: Area under the curve
WB: Western blotting
IHC: Immunohistochemistry
