# Supplementary material for: Causal role of MiRNAs in chronic rhinosinusitis: mendelian randomization and validation study
Source: Allergy Asthma Clin Immunol. 2025 Apr 17;21:17. doi: 10.1186/s13223-025-00957-4 (PMC12007379; doi:10.1186/s13223-025-00957-4)
Supplement: Supplementary file 1 — Supplementary Material 1 [file 13223_2025_957_MOESM1_ESM.docx]

**Supplementary Table S1.** Summary of patient’s characteristics.

| Variable | Control | CRSwNP | P-value |
| --- | --- | --- | --- |
| Total of patient,n | 14 | 32 |  |
| Age(yr), median (IQR) | 50.00 (41.00, 57.75) | 49.50 (37.50, 55.25) | 0.481 |
| Female,n(%) | 6(42.86) | 15(46.88) | 0.801 |
| BMI(kg/m²), median (IQR) | 25.05 (24.33, 25.68) | 22.40 (21.18, 25.38) | 0.088 |
| Smoker,n(%) | 4(28.57) | 10(31.25) | 0.869 |
| qRT-PCR | 14 | 32 |  |
| Age(yr), median (IQR) | 50.00 (41.00, 57.75) | 49.50 (37.50, 55.25) | 0.481 |
| WB | 6 | 6 |  |
| Age(yr), mean±SD | 47.33 ± 14.15 | 41.67 ± 10.09 | 0.445 |
| IHC | 4 | 4 |  |
| Age(yr), mean±SD | 51.75 ± 16.82 | 46.75 ± 14.97 | 0.673 |

BMI, Body Mass Index; qRT-PCR, Quantitative real-time PCR; WB, Western Blot; IHC, Immunohistochemistry

**Supplementary Table S2**. Primers used for real-time PCR analysis of microRNAs.

| **Primer** | **Sequence** |
| --- | --- |
| hsa-miR-130a-3p  stem-loop RT | 5’-GTCGTATCCAGTGCAGGGTCCGAGGTATTCGCACTGGATACGACATGCCC-3’ |
| hsa-miR-130a-3p  PCR Forward | 5’-CCACGGAGCAGTGCAATGTTAAA-3’ |
| hsa-miR-196b-5p  stem-loop RT | 5’-GTCGTATCCAGTGCAGGGTCCGAGGTATTCGCACTGGATACGACCCCAAC-3’ |
| hsa-miR-196b-5p  PCR Forward | 5’-GCGCGTAGGTAGTTTCCTGTT-3’ |
| hsa-miR-339-3p  stem-loop RT | 5’-GTCGTATCCAGTGCAGGGTCCGAGGTATTCGCACTGGATACGACCGGCTC-3’ |
| hsa-miR-339-3p  PCR Forward | 5’-AAGAATTTGAGCGCCTCGACGA-3’ |
| U6 stem-loop RT | 5’-GTCGTATCCAGTGCAGGGTCCGAGGTATTCGCACTGGATACGACAAAATATG-3’ |
| U6 PCR Forward | 5’-CTCGCTTCGGCAGCACA-3’ |
| microRNA PCR universal Reverse | 5’-ATCCAGTGCAGGGTCCGAGG-3’ |

**Supplementary Table S3.**The list of Antibody

| Antibody | Cat No. | Vendor | Application |
| --- | --- | --- | --- |
| PIK3CA | 67071-1-Ig | proteintech | IHC,1:500 |
| P-Pi3k | 341468 | zenbio | IHC,1:50 |
| P-AKT | 4060 | Cell Signaling | WB,1:1000 |
| AKT | 60203-2-Ig | proteintech | WB,1:5000 |
| GADPH | 10494-1-AP | proteintech | WB,1:10000 |
| P38 | bs-0637R | Bioss | WB,1:500 |
| P-P38 | bs-5476R | Bioss | WB,1:500; ICH,1:200 |
| ERK | 4695 | Cell Signaling | WB,1:1000 |
| P-ERK | 4370 | Cell Signaling | WB,1:1000 |
| JNK | 9252 | Cell Signaling | WB,1:1000 |
| P-JNK | 9251 | Cell Signaling | WB,1:1000 |

**Supplementary Table S4.** Characteristics of genetic variants used to estimate the effect of miRNAs on chronic sinusitis in the Ebi discovery cohort

| **SNP** | **chr** | **pos** | **A1** | **A2** | **R^2^** | **F** | **miR-130a-3p** | | | **Ebi chronic sinusitis** | | |
| --- | --- | --- | --- | --- | --- | --- | --- | --- | --- | --- | --- | --- |
|  |  |  |  |  |  |  | **beta** | **se** | **P-value** | **beta** | **se** | **P-value** |
| rs10896647 | 11 | 57571883 | T | C | 0.0022 | 25.0430 | 0.3088 | 0.0617 | 5.80E-07 | 0.0072 | 0.0159 | 6.50E-01 |
| rs10896661 | 11 | 57679029 | A | C | 0.0022 | 25.5012 | 0.3315 | 0.0656 | 4.58E-07 | 0.0137 | 0.0167 | 4.12E-01 |
| rs11512732 | 11 | 57756568 | G | A | 0.0020 | 21.0001 | 0.2709 | 0.0591 | 4.70E-06 | 0.0181 | 0.0162 | 2.64E-01 |
| rs11570190 | 11 | 57560452 | C | A | 0.0022 | 25.1671 | 0.3084 | 0.0615 | 5.44E-07 | 0.0070 | 0.0159 | 6.60E-01 |
| rs11601320 | 11 | 57642617 | C | T | 0.0021 | 24.7725 | 0.3088 | 0.0620 | 6.66E-07 | 0.0086 | 0.0160 | 5.93E-01 |
| rs11603026 | 11 | 57873888 | T | C | 0.0002 | 17.0234 | 0.9539 | 0.2312 | 3.75E-05 | 0.0654 | 0.0441 | 1.39E-01 |
| rs11606677 | 11 | 57383377 | A | G | 0.0022 | 28.0551 | 0.3421 | 0.0646 | 1.23E-07 | 0.0137 | 0.0166 | 4.11E-01 |
| rs12798206 | 11 | 57668479 | G | T | 0.0022 | 25.9386 | 0.3251 | 0.0638 | 3.65E-07 | 0.0143 | 0.0167 | 3.89E-01 |
| rs12799639 | 11 | 57757561 | T | C | 0.0016 | 18.5733 | 0.2676 | 0.0621 | 1.67E-05 | 0.0183 | 0.0143 | 2.02E-01 |
| rs12804093 | 11 | 57734088 | C | T | 0.0017 | 19.6828 | 0.2762 | 0.0623 | 9.34E-06 | 0.0293 | 0.0160 | 6.71E-02 |
| rs12807250 | 11 | 57600285 | T | C | 0.0021 | 24.8413 | 0.3084 | 0.0619 | 6.43E-07 | 0.0068 | 0.0159 | 6.72E-01 |
| rs2441952 | 11 | 57792001 | C | T | 0.0017 | 19.9082 | 0.2747 | 0.0616 | 8.30E-06 | 0.0173 | 0.0143 | 2.25E-01 |
| rs2443441 | 11 | 57903862 | C | A | 0.0022 | 23.8323 | 0.2935 | 0.0601 | 1.08E-06 | 0.0256 | 0.0137 | 6.19E-02 |
| rs2454663 | 11 | 57358083 | C | T | 0.0014 | 19.9077 | 0.3263 | 0.0731 | 8.30E-06 | -0.0086 | 0.0167 | 6.04E-01 |
| rs2511983 | 11 | 57346874 | G | A | 0.0011 | 16.4450 | 0.2842 | 0.0701 | 5.08E-05 | -0.0097 | 0.0166 | 5.61E-01 |
| rs2649665 | 11 | 57289328 | C | A | 0.0014 | 16.3891 | 0.3153 | 0.0779 | 5.24E-05 | 0.0211 | 0.0343 | 5.38E-01 |
| rs35808061 | 11 | 57547000 | C | T | 0.0023 | 26.7309 | 0.3220 | 0.0623 | 2.43E-07 | 0.0070 | 0.0159 | 6.59E-01 |
| rs4272798 | 11 | 57743634 | T | C | 0.0018 | 19.3689 | 0.2639 | 0.0600 | 1.10E-05 | 0.0182 | 0.0162 | 2.61E-01 |
| rs4939180 | 11 | 57863416 | T | C | 0.0022 | 24.2742 | 0.2918 | 0.0592 | 8.62E-07 | 0.0319 | 0.0137 | 2.02E-02 |
| rs499188 | 11 | 57434122 | A | C | 0.0021 | 24.1823 | 0.3043 | 0.0619 | 9.04E-07 | 0.0065 | 0.0160 | 6.85E-01 |
| rs547891 | 11 | 57499065 | C | T | 0.0021 | 24.5056 | 0.3047 | 0.0615 | 7.65E-07 | 0.0063 | 0.0159 | 6.95E-01 |
| rs6591444 | 11 | 57724059 | A | G | 0.0017 | 19.5948 | 0.2755 | 0.0622 | 9.77E-06 | 0.0323 | 0.0162 | 4.68E-02 |
| rs685149 | 11 | 57657413 | A | G | 0.0026 | 29.1709 | 0.3276 | 0.0607 | 6.93E-08 | 0.0113 | 0.0156 | 4.70E-01 |
| rs708228 | 11 | 57585662 | T | C | 0.0021 | 24.9312 | 0.3086 | 0.0618 | 6.14E-07 | 0.0074 | 0.0159 | 6.41E-01 |
| rs7102271 | 11 | 57396825 | T | G | 0.0022 | 27.6178 | 0.3361 | 0.0640 | 1.54E-07 | 0.0124 | 0.0167 | 4.57E-01 |
| rs7117878 | 11 | 57421457 | A | C | 0.0023 | 27.2549 | 0.3339 | 0.0640 | 1.85E-07 | 0.0052 | 0.0161 | 7.46E-01 |
| rs7121169 | 11 | 57452543 | A | G | 0.0021 | 24.2648 | 0.3046 | 0.0618 | 8.66E-07 | 0.0071 | 0.0160 | 6.58E-01 |
| rs731384 | 11 | 57408382 | A | G | 0.0021 | 26.0883 | 0.3410 | 0.0668 | 3.38E-07 | 0.0146 | 0.0167 | 3.82E-01 |
| rs895660 | 11 | 57878487 | A | G | 0.0023 | 25.0720 | 0.3044 | 0.0608 | 5.71E-07 | 0.0316 | 0.0137 | 2.12E-02 |
| rs9420 | 11 | 57510294 | A | G | 0.0021 | 24.7987 | 0.3073 | 0.0617 | 6.58E-07 | 0.0044 | 0.0160 | 7.82E-01 |
| **SNP** | **chr** | **pos** | **A1** | **A2** | **R^2^** | **F** | **miR-196b-5p** | | | **Ebi chronic sinusitis** | | |
|  |  |  |  |  |  |  | **beta** | **se** | **P-value** | **beta** | **se** | **P-value** |
| rs10085570 | 7 | 27151188 | A | G | 0.0010 | 22.4054 | 0.2778 | 0.0587 | 2.28E-06 | 0.0449 | 0.0185 | 1.52E-02 |
| rs10228276 | 7 | 27247279 | A | G | 0.0019 | 25.9949 | 0.2385 | 0.0468 | 3.58E-07 | -0.0002 | 0.0153 | 9.90E-01 |
| rs10233387 | 7 | 27243106 | A | G | 0.0045 | 37.5213 | 0.2312 | 0.0377 | 9.88E-10 | 0.0142 | 0.0134 | 2.91E-01 |
| rs10246712 | 7 | 27198860 | G | A | 0.0017 | 33.1490 | 0.3289 | 0.0571 | 9.15E-09 | 0.0455 | 0.0182 | 1.23E-02 |
| rs10257464 | 7 | 27065778 | A | C | 0.0014 | 23.6856 | 0.2497 | 0.0513 | 1.18E-06 | 0.0327 | 0.0252 | 1.94E-01 |
| rs10951154 | 7 | 27135314 | T | C | 0.0017 | 26.7114 | 0.3257 | 0.0630 | 2.47E-07 | 0.0206 | 0.0419 | 6.23E-01 |
| rs11983200 | 7 | 27186596 | A | G | 0.0015 | 30.7937 | 0.3144 | 0.0567 | 3.05E-08 | 0.0435 | 0.0199 | 2.88E-02 |
| rs12533947 | 7 | 27175295 | A | G | 0.0015 | 20.1765 | 0.2665 | 0.0593 | 7.25E-06 | 0.0344 | 0.0169 | 4.20E-02 |
| rs13221446 | 7 | 27094976 | G | T | 0.0014 | 23.8818 | 0.2520 | 0.0516 | 1.06E-06 | 0.0328 | 0.0393 | 4.04E-01 |
| rs1725074 | 7 | 27144921 | C | T | 0.0010 | 19.1051 | 0.2855 | 0.0653 | 1.27E-05 | 0.0295 | 0.0187 | 1.15E-01 |
| rs1859164 | 7 | 27218419 | C | T | 0.0068 | 59.3656 | 0.3904 | 0.0507 | 1.63E-14 | 0.0374 | 0.0134 | 5.31E-03 |
| rs1859601 | 7 | 26590857 | T | C | 0.0017 | 22.5382 | 0.2121 | 0.0447 | 2.13E-06 | 0.0155 | 0.0169 | 3.59E-01 |
| rs2071243 | 7 | 27208280 | T | C | 0.0070 | 60.2335 | 0.4010 | 0.0517 | 1.05E-14 | 0.0384 | 0.0134 | 4.06E-03 |
| rs213521 | 7 | 26942835 | C | T | 0.0012 | 20.5358 | 0.2298 | 0.0507 | 6.02E-06 | 0.0339 | 0.0250 | 1.75E-01 |
| rs2140998 | 7 | 27026872 | A | C | 0.0014 | 23.9530 | 0.2507 | 0.0512 | 1.02E-06 | 0.0316 | 0.0252 | 2.09E-01 |
| rs2189239 | 7 | 27237453 | C | T | 0.0008 | 17.8090 | 0.2536 | 0.0601 | 2.49E-05 | 0.0824 | 0.0345 | 1.70E-02 |
| rs2285724 | 7 | 27227359 | A | G | 0.0040 | 34.4457 | 0.2266 | 0.0386 | 4.73E-09 | 0.0151 | 0.0135 | 2.64E-01 |
| rs3801776 | 7 | 27205282 | G | A | 0.0029 | 32.0929 | 0.2927 | 0.0517 | 1.57E-08 | 0.0258 | 0.0147 | 7.90E-02 |
| rs4552808 | 7 | 26607584 | T | C | 0.0017 | 21.7152 | 0.2080 | 0.0446 | 3.26E-06 | 0.0168 | 0.0169 | 3.23E-01 |
| rs706017 | 7 | 27139878 | A | G | 0.0009 | 22.4627 | 0.3122 | 0.0659 | 2.21E-06 | 0.0638 | 0.0331 | 5.39E-02 |
| rs774246 | 7 | 26990816 | A | G | 0.0012 | 21.3127 | 0.2336 | 0.0506 | 4.02E-06 | 0.0372 | 0.0250 | 1.36E-01 |
| rs774257 | 7 | 26977192 | G | A | 0.0012 | 20.7059 | 0.2305 | 0.0507 | 5.51E-06 | 0.0348 | 0.0251 | 1.66E-01 |
| rs7810502 | 7 | 27203139 | A | G | 0.0026 | 23.9426 | 0.2466 | 0.0504 | 1.03E-06 | 0.0332 | 0.0140 | 1.79E-02 |
| rs875896 | 7 | 27201532 | A | C | 0.0021 | 26.3745 | 0.2292 | 0.0446 | 2.94E-07 | 0.0185 | 0.0147 | 2.06E-01 |
| rs983186 | 7 | 27188659 | A | G | 0.0024 | 32.0876 | 0.2922 | 0.0516 | 1.57E-08 | 0.0186 | 0.0148 | 2.07E-01 |
| **SNP** | **chr** | **pos** | **A1** | **A2** | **R^2^** | **F** | **miR-339-3p** | | | **Ebi chronic sinusitis** | | |
|  |  |  |  |  |  |  | **beta** | **se** | **P-value** | **beta** | **se** | **P-value** |
| rs10224368 | 7 | 1081972 | A | C | 0.0058 | 64.6018 | 0.2969 | 0.0369 | 1.13E-15 | -0.0005 | 0.0142 | 9.71E-01 |
| rs10252234 | 7 | 1114381 | T | C | 0.0085 | 130.1887 | 0.4963 | 0.0435 | 8.42E-30 | -0.0237 | 0.0194 | 2.22E-01 |
| rs10252404 | 7 | 1209607 | A | C | 0.0069 | 133.2497 | 0.5731 | 0.0496 | 1.87E-30 | -0.0142 | 0.0241 | 5.56E-01 |
| rs10257328 | 7 | 1092886 | G | T | 0.0072 | 74.5178 | 0.4523 | 0.0524 | 7.89E-18 | -0.0117 | 0.0146 | 4.22E-01 |
| rs10265736 | 7 | 1172465 | T | C | 0.0078 | 140.7071 | 0.5479 | 0.0462 | 4.82E-32 | -0.0202 | 0.0228 | 3.76E-01 |
| rs10266519 | 7 | 1142977 | T | C | 0.0081 | 147.1898 | 0.5661 | 0.0467 | 2.01E-33 | -0.0271 | 0.0228 | 2.35E-01 |
| rs10274964 | 7 | 1019784 | C | T | 0.0018 | 19.6723 | 0.1727 | 0.0389 | 9.38E-06 | 0.0106 | 0.0138 | 4.42E-01 |
| rs10278051 | 7 | 1181139 | T | C | 0.0049 | 55.9981 | 0.2969 | 0.0397 | 8.47E-14 | 0.0024 | 0.0145 | 8.69E-01 |
| rs10951499 | 7 | 1065746 | G | A | 0.0062 | 69.0932 | 0.3082 | 0.0371 | 1.19E-16 | -0.0085 | 0.0142 | 5.48E-01 |
| rs1104888 | 7 | 1106012 | G | T | 0.0097 | 100.7732 | 0.3868 | 0.0385 | 1.69E-23 | -0.0041 | 0.0143 | 7.76E-01 |
| rs1133122 | 7 | 1192572 | A | C | 0.0076 | 136.3766 | 0.5331 | 0.0456 | 4.03E-31 | -0.0250 | 0.0227 | 2.71E-01 |
| rs11559183 | 7 | 1037025 | A | C | 0.0023 | 69.7928 | 0.5118 | 0.0613 | 8.37E-17 | -0.0531 | 0.0317 | 9.38E-02 |
| rs11761037 | 7 | 1126535 | G | A | 0.0015 | 64.3603 | 0.6211 | 0.0774 | 1.27E-15 | 0.0206 | 0.0366 | 5.73E-01 |
| rs11763020 | 7 | 1060288 | T | C | 0.0075 | 146.7557 | 0.5932 | 0.0490 | 2.49E-33 | -0.0248 | 0.0247 | 3.15E-01 |
| rs11763793 | 7 | 1094342 | G | A | 0.0079 | 154.4576 | 0.5978 | 0.0481 | 5.76E-35 | -0.0339 | 0.0248 | 1.71E-01 |
| rs11974176 | 7 | 1065569 | T | C | 0.0080 | 91.7664 | 0.3779 | 0.0395 | 1.47E-21 | -0.0007 | 0.0143 | 9.62E-01 |
| rs12056053 | 7 | 1197632 | C | A | 0.0071 | 74.7265 | 0.3226 | 0.0373 | 7.11E-18 | -0.0148 | 0.0135 | 2.73E-01 |
| rs12381396 | 7 | 1046777 | G | A | 0.0012 | 37.8026 | 0.4616 | 0.0751 | 8.41E-10 | 0.0050 | 0.0184 | 7.87E-01 |
| rs12701834 | 7 | 1105426 | G | A | 0.0091 | 105.4188 | 0.4065 | 0.0396 | 1.69E-24 | 0.0010 | 0.0157 | 9.47E-01 |
| rs12702016 | 7 | 1129392 | T | C | 0.0047 | 52.1727 | 0.2657 | 0.0368 | 5.82E-13 | -0.0106 | 0.0136 | 4.34E-01 |
| rs12702029 | 7 | 1130584 | C | T | 0.0017 | 17.4889 | 0.1730 | 0.0414 | 2.94E-05 | 0.0040 | 0.0133 | 7.64E-01 |
| rs13232402 | 7 | 1055598 | A | G | 0.0030 | 38.3482 | 0.2446 | 0.0395 | 6.37E-10 | -0.0149 | 0.0146 | 3.08E-01 |
| rs17234336 | 7 | 1220005 | T | C | 0.0014 | 52.9026 | 0.8496 | 0.1168 | 4.03E-13 | 0.0158 | 0.0359 | 6.59E-01 |
| rs1997243 | 7 | 1083777 | G | A | 0.0078 | 152.9682 | 0.5940 | 0.0480 | 1.19E-34 | -0.0277 | 0.0248 | 2.64E-01 |
| rs2070118 | 7 | 1132505 | A | G | 0.0079 | 111.8326 | 0.4289 | 0.0406 | 7.12E-26 | -0.0261 | 0.0201 | 1.94E-01 |
| rs3735686 | 7 | 1062527 | A | G | 0.0039 | 41.2351 | 0.2355 | 0.0367 | 1.47E-10 | -0.0013 | 0.0134 | 9.22E-01 |
| rs3824080 | 7 | 939324 | T | C | 0.0006 | 16.5950 | 2.8142 | 0.6908 | 4.70E-05 | -0.0457 | 0.0177 | 1.00E-02 |
| rs4722935 | 7 | 994621 | C | T | 0.0013 | 21.2815 | 0.6289 | 0.1363 | 4.06E-06 | -0.0276 | 0.0163 | 9.11E-02 |
| rs4724104 | 7 | 1120918 | C | T | 0.0018 | 32.7551 | 0.5754 | 0.1005 | 1.10E-08 | 0.0001 | 0.0169 | 9.96E-01 |
| rs4724463 | 7 | 1160190 | G | A | 0.0067 | 87.7125 | 0.3668 | 0.0392 | 1.10E-20 | -0.0180 | 0.0191 | 3.47E-01 |
| rs6945202 | 7 | 1116558 | C | T | 0.0078 | 82.2461 | 0.3500 | 0.0386 | 1.67E-19 | 0.0018 | 0.0134 | 8.94E-01 |
| rs6947257 | 7 | 1046906 | C | T | 0.0041 | 44.3119 | 0.2516 | 0.0378 | 3.09E-11 | 0.0014 | 0.0136 | 9.16E-01 |
| rs6952546 | 7 | 988009 | A | G | 0.0066 | 136.1030 | 1.5070 | 0.1292 | 4.61E-31 | -0.0293 | 0.0252 | 2.44E-01 |
| rs7784043 | 7 | 1210554 | G | T | 0.0062 | 65.5545 | 0.3002 | 0.0371 | 6.98E-16 | 0.0198 | 0.0139 | 1.55E-01 |
| rs7784607 | 7 | 1178694 | G | A | 0.0057 | 62.7349 | 0.2999 | 0.0379 | 2.87E-15 | -0.0128 | 0.0136 | 3.49E-01 |
| rs7787534 | 7 | 1225076 | C | A | 0.0018 | 22.4494 | 0.2961 | 0.0625 | 2.22E-06 | -0.0263 | 0.0150 | 7.98E-02 |
| rs7807150 | 7 | 1020272 | A | G | 0.0022 | 51.7302 | 0.4001 | 0.0556 | 7.27E-13 | -0.0097 | 0.0221 | 6.62E-01 |
| rs7809203 | 7 | 1087454 | C | T | 0.0058 | 60.9918 | 0.2802 | 0.0359 | 6.89E-15 | 0.0010 | 0.0133 | 9.40E-01 |
| rs9640009 | 7 | 1231861 | T | C | 0.0017 | 18.1666 | 0.1650 | 0.0387 | 2.06E-05 | -0.0131 | 0.0138 | 3.45E-01 |
| rs9655470 | 7 | 1151713 | T | G | 0.0072 | 74.8350 | 0.3065 | 0.0354 | 6.73E-18 | -0.0110 | 0.0132 | 4.06E-01 |

SNP, single nucleotide polymorphism; A1, effect allele; A2, other allele; se, standard error.

**Supplementary Table S5.** Characteristics of genetic variants used to estimate the effect of miRNAs on chronic sinusitis in the UKB replication cohort

| **SNP** | **chr** | **pos** | **A1** | **A2** | **R^2^** | **F** | **miR-130a-3p** | | | | | **UKB chronic sinusitis** | | | | | |
| --- | --- | --- | --- | --- | --- | --- | --- | --- | --- | --- | --- | --- | --- | --- | --- | --- | --- |
|  |  |  |  |  |  |  | **beta** | **se** | | **P-value** | | **beta** | **se** | | | **P-value** | |
| rs10896647 | 11 | 57571883 | T | C | 0.0022 | 25.0430 | 0.3088 | | 0.0617 | | 5.80E-07 | 0.0002 | | 0.0001 | | | 2.40E-01 |
| rs10896661 | 11 | 57679029 | A | C | 0.0022 | 25.5012 | 0.3315 | | 0.0656 | | 4.58E-07 | 0.0002 | | 0.0001 | | | 1.90E-01 |
| rs11512732 | 11 | 57756568 | G | A | 0.0020 | 21.0001 | 0.2709 | | 0.0591 | | 4.70E-06 | 0.0003 | | 0.0001 | | | 2.51E-02 |
| rs11570190 | 11 | 57560452 | C | A | 0.0022 | 25.1671 | 0.3084 | | 0.0615 | | 5.44E-07 | 0.0002 | | 0.0001 | | | 2.46E-01 |
| rs11601320 | 11 | 57642617 | C | T | 0.0021 | 24.7725 | 0.3088 | | 0.0620 | | 6.66E-07 | 0.0002 | | 0.0001 | | | 2.94E-01 |
| rs11606677 | 11 | 57383377 | A | G | 0.0022 | 28.0551 | 0.3421 | | 0.0646 | | 1.23E-07 | 0.0002 | | 0.0002 | | | 2.49E-01 |
| rs12798206 | 11 | 57668479 | G | T | 0.0022 | 25.9386 | 0.3251 | | 0.0638 | | 3.65E-07 | 0.0002 | | 0.0001 | | | 1.90E-01 |
| rs12799639 | 11 | 57757561 | T | C | 0.0016 | 18.5733 | 0.2676 | | 0.0621 | | 1.67E-05 | 0.0002 | | 0.0001 | | | 1.54E-01 |
| rs12804093 | 11 | 57734088 | C | T | 0.0017 | 19.6828 | 0.2762 | | 0.0623 | | 9.34E-06 | 0.0002 | | 0.0001 | | | 1.84E-01 |
| rs12807250 | 11 | 57600285 | T | C | 0.0021 | 24.8413 | 0.3084 | | 0.0619 | | 6.43E-07 | 0.0002 | | 0.0001 | | | 2.27E-01 |
| rs2441952 | 11 | 57792001 | C | T | 0.0017 | 19.9082 | 0.2747 | | 0.0616 | | 8.30E-06 | 0.0002 | | 0.0001 | | | 2.42E-01 |
| rs2443441 | 11 | 57903862 | C | A | 0.0022 | 23.8323 | 0.2935 | | 0.0601 | | 1.08E-06 | 0.0002 | | 0.0001 | | | 1.11E-01 |
| rs2454663 | 11 | 57358083 | C | T | 0.0014 | 19.9077 | 0.3263 | | 0.0731 | | 8.30E-06 | 0.0000 | | 0.0002 | | | 7.90E-01 |
| rs2511983 | 11 | 57346874 | G | A | 0.0011 | 16.4450 | 0.2842 | | 0.0701 | | 5.08E-05 | 0.0000 | | 0.0002 | | | 8.23E-01 |
| rs2649665 | 11 | 57289328 | C | A | 0.0014 | 16.3891 | 0.3153 | | 0.0779 | | 5.24E-05 | 0.0000 | | 0.0002 | | | 9.83E-01 |
| rs35808061 | 11 | 57547000 | C | T | 0.0023 | 26.7309 | 0.3220 | | 0.0623 | | 2.43E-07 | 0.0002 | | 0.0001 | | | 2.46E-01 |
| rs4272798 | 11 | 57743634 | T | C | 0.0018 | 19.3689 | 0.2639 | | 0.0600 | | 1.10E-05 | 0.0003 | | 0.0001 | | | 2.89E-02 |
| rs4939180 | 11 | 57863416 | T | C | 0.0022 | 24.2742 | 0.2918 | | 0.0592 | | 8.62E-07 | 0.0003 | | 0.0001 | | | 6.85E-02 |
| rs499188 | 11 | 57434122 | A | C | 0.0021 | 24.1823 | 0.3043 | | 0.0619 | | 9.04E-07 | 0.0002 | | 0.0001 | | | 2.37E-01 |
| rs547891 | 11 | 57499065 | C | T | 0.0021 | 24.5056 | 0.3047 | | 0.0615 | | 7.65E-07 | 0.0002 | | 0.0001 | | | 2.83E-01 |
| rs6591444 | 11 | 57724059 | A | G | 0.0017 | 19.5948 | 0.2755 | | 0.0622 | | 9.77E-06 | 0.0002 | | 0.0001 | | | 1.72E-01 |
| rs685149 | 11 | 57657413 | A | G | 0.0026 | 29.1709 | 0.3276 | | 0.0607 | | 6.93E-08 | 0.0002 | | 0.0001 | | | 8.48E-02 |
| rs708228 | 11 | 57585662 | T | C | 0.0021 | 24.9312 | 0.3086 | | 0.0618 | | 6.14E-07 | 0.0002 | | 0.0001 | | | 2.44E-01 |
| rs7102271 | 11 | 57396825 | T | G | 0.0022 | 27.6178 | 0.3361 | | 0.0640 | | 1.54E-07 | 0.0002 | | 0.0002 | | | 2.64E-01 |
| rs7117878 | 11 | 57421457 | A | C | 0.0023 | 27.2549 | 0.3339 | | 0.0640 | | 1.85E-07 | 0.0002 | | 0.0001 | | | 2.22E-01 |
| rs7121169 | 11 | 57452543 | A | G | 0.0021 | 24.2648 | 0.3046 | | 0.0618 | | 8.66E-07 | 0.0001 | | 0.0001 | | | 3.07E-01 |
| rs731384 | 11 | 57408382 | A | G | 0.0021 | 26.0883 | 0.3410 | | 0.0668 | | 3.38E-07 | 0.0001 | | 0.0002 | | | 3.37E-01 |
| rs895660 | 11 | 57878487 | A | G | 0.0023 | 25.0720 | 0.3044 | | 0.0608 | | 5.71E-07 | 0.0003 | | 0.0001 | | | 8.34E-02 |
| rs9420 | 11 | 57510294 | A | G | 0.0021 | 24.7987 | 0.3073 | | 0.0617 | | 6.58E-07 | 0.0002 | | 0.0001 | | | 2.81E-01 |
| **SNP** | **chr** | **pos** | **A1** | **A2** | **R^2^** | **F** | **miR-196b-5p** | | | | | **Ebi chronic sinusitis** | | | | | |
|  |  |  |  |  |  |  | **beta** | **se** | | **P-value** | | **beta** | **se** | | | **P-value** | |
| rs10085570 | 7 | 27151188 | A | G | 0.0010 | 22.4054 | 0.2778 | | 0.0587 | | 2.28E-06 | 0.0003 | | 0.0002 | | | 1.33E-01 |
| rs10228276 | 7 | 27247279 | A | G | 0.0019 | 25.9949 | 0.2385 | | 0.0468 | | 3.58E-07 | 0.0003 | | 0.0002 | | | 7.83E-02 |
| rs10233387 | 7 | 27243106 | A | G | 0.0045 | 37.5213 | 0.2312 | | 0.0377 | | 9.88E-10 | 0.0004 | | 0.0001 | | | 8.39E-03 |
| rs10246712 | 7 | 27198860 | G | A | 0.0017 | 33.1490 | 0.3289 | | 0.0571 | | 9.15E-09 | 0.0003 | | 0.0002 | | | 1.25E-01 |
| rs10257464 | 7 | 27065778 | A | C | 0.0014 | 23.6856 | 0.2497 | | 0.0513 | | 1.18E-06 | 0.0001 | | 0.0002 | | | 5.66E-01 |
| rs10951154 | 7 | 27135314 | T | C | 0.0017 | 26.7114 | 0.3257 | | 0.0630 | | 2.47E-07 | 0.0001 | | 0.0002 | | | 6.00E-01 |
| rs11983200 | 7 | 27186596 | A | G | 0.0015 | 30.7937 | 0.3144 | | 0.0567 | | 3.05E-08 | 0.0003 | | 0.0002 | | | 2.49E-01 |
| rs12533947 | 7 | 27175295 | A | G | 0.0015 | 20.1765 | 0.2665 | | 0.0593 | | 7.25E-06 | 0.0003 | | 0.0002 | | | 6.62E-02 |
| rs13221446 | 7 | 27094976 | G | T | 0.0014 | 23.8818 | 0.2520 | | 0.0516 | | 1.06E-06 | 0.0001 | | 0.0002 | | | 6.00E-01 |
| rs1725074 | 7 | 27144921 | C | T | 0.0010 | 19.1051 | 0.2855 | | 0.0653 | | 1.27E-05 | 0.0000 | | 0.0002 | | | 9.57E-01 |
| rs1859164 | 7 | 27218419 | C | T | 0.0068 | 59.3656 | 0.3904 | | 0.0507 | | 1.63E-14 | 0.0003 | | 0.0001 | | | 3.91E-02 |
| rs1859601 | 7 | 26590857 | T | C | 0.0017 | 22.5382 | 0.2121 | | 0.0447 | | 2.13E-06 | -0.0001 | | 0.0002 | | | 6.63E-01 |
| rs2071243 | 7 | 27208280 | T | C | 0.0070 | 60.2335 | 0.4010 | | 0.0517 | | 1.05E-14 | 0.0003 | | 0.0001 | | | 2.67E-02 |
| rs213521 | 7 | 26942835 | C | T | 0.0012 | 20.5358 | 0.2298 | | 0.0507 | | 6.02E-06 | 0.0001 | | 0.0002 | | | 6.07E-01 |
| rs2140998 | 7 | 27026872 | A | C | 0.0014 | 23.9530 | 0.2507 | | 0.0512 | | 1.02E-06 | 0.0001 | | 0.0002 | | | 6.68E-01 |
| rs2189239 | 7 | 27237453 | C | T | 0.0008 | 17.8090 | 0.2536 | | 0.0601 | | 2.49E-05 | 0.0005 | | 0.0003 | | | 6.43E-02 |
| rs2285724 | 7 | 27227359 | A | G | 0.0040 | 34.4457 | 0.2266 | | 0.0386 | | 4.73E-09 | 0.0003 | | 0.0001 | | | 2.57E-02 |
| rs3801776 | 7 | 27205282 | G | A | 0.0029 | 32.0929 | 0.2927 | | 0.0517 | | 1.57E-08 | 0.0002 | | 0.0002 | | | 2.52E-01 |
| rs4552808 | 7 | 26607584 | T | C | 0.0017 | 21.7152 | 0.2080 | | 0.0446 | | 3.26E-06 | 0.0000 | | 0.0002 | | | 7.89E-01 |
| rs706017 | 7 | 27139878 | A | G | 0.0009 | 22.4627 | 0.3122 | | 0.0659 | | 2.21E-06 | 0.0003 | | 0.0002 | | | 2.37E-01 |
| rs774246 | 7 | 26990816 | A | G | 0.0012 | 21.3127 | 0.2336 | | 0.0506 | | 4.02E-06 | 0.0001 | | 0.0002 | | | 7.57E-01 |
| rs774257 | 7 | 26977192 | G | A | 0.0012 | 20.7059 | 0.2305 | | 0.0507 | | 5.51E-06 | 0.0001 | | 0.0002 | | | 6.40E-01 |
| rs7810502 | 7 | 27203139 | A | G | 0.0026 | 23.9426 | 0.2466 | | 0.0504 | | 1.03E-06 | 0.0001 | | 0.0002 | | | 3.65E-01 |
| rs875896 | 7 | 27201532 | A | C | 0.0021 | 26.3745 | 0.2292 | | 0.0446 | | 2.94E-07 | 0.0001 | | 0.0002 | | | 4.48E-01 |
| rs983186 | 7 | 27188659 | A | G | 0.0024 | 32.0876 | 0.2922 | | 0.0516 | | 1.57E-08 | 0.0001 | | 0.0002 | | | 4.86E-01 |
| **SNP** | **chr** | **pos** | **A1** | **A2** | **R^2^** | **F** | **miR-339-3p** | | | | | **Ebi chronic sinusitis** | | | | | |
|  |  |  |  |  |  |  | **beta** | | **se** | | **P-value** | **beta** | | | **se** | | **P-value** |
| rs10224368 | 7 | 1081972 | A | C | 0.0058 | 64.6018 | 0.2969 | | 0.0369 | | 1.13E-15 | -0.0002 | | 0.0001 | | | 2.89E-01 |
| rs10252234 | 7 | 1114381 | T | C | 0.0085 | 130.1887 | 0.4963 | | 0.0435 | | 8.42E-30 | -0.0002 | | 0.0002 | | | 1.87E-01 |
| rs10252404 | 7 | 1209607 | A | C | 0.0069 | 133.2497 | 0.5731 | | 0.0496 | | 1.87E-30 | -0.0003 | | 0.0002 | | | 1.64E-01 |
| rs10257328 | 7 | 1092886 | G | T | 0.0072 | 74.5178 | 0.4523 | | 0.0524 | | 7.89E-18 | -0.0002 | | 0.0001 | | | 1.80E-01 |
| rs10265736 | 7 | 1172465 | T | C | 0.0078 | 140.7071 | 0.5479 | | 0.0462 | | 4.82E-32 | -0.0003 | | 0.0002 | | | 8.85E-02 |
| rs10266519 | 7 | 1142977 | T | C | 0.0081 | 147.1898 | 0.5661 | | 0.0467 | | 2.01E-33 | -0.0003 | | 0.0002 | | | 1.28E-01 |
| rs10274964 | 7 | 1019784 | C | T | 0.0018 | 19.6723 | 0.1727 | | 0.0389 | | 9.38E-06 | -0.0001 | | 0.0001 | | | 5.25E-01 |
| rs10278051 | 7 | 1181139 | T | C | 0.0049 | 55.9981 | 0.2969 | | 0.0397 | | 8.47E-14 | -0.0002 | | 0.0001 | | | 2.39E-01 |
| rs10951499 | 7 | 1065746 | G | A | 0.0062 | 69.0932 | 0.3082 | | 0.0371 | | 1.19E-16 | -0.0001 | | 0.0001 | | | 5.69E-01 |
| rs1104888 | 7 | 1106012 | G | T | 0.0097 | 100.7732 | 0.3868 | | 0.0385 | | 1.69E-23 | 0.0000 | | 0.0001 | | | 9.74E-01 |
| rs1133122 | 7 | 1192572 | A | C | 0.0076 | 136.3766 | 0.5331 | | 0.0456 | | 4.03E-31 | -0.0003 | | 0.0002 | | | 5.94E-02 |
| rs11559183 | 7 | 1037025 | A | C | 0.0023 | 69.7928 | 0.5118 | | 0.0613 | | 8.37E-17 | -0.0001 | | 0.0002 | | | 6.52E-01 |
| rs11761037 | 7 | 1126535 | G | A | 0.0015 | 64.3603 | 0.6211 | | 0.0774 | | 1.27E-15 | -0.0004 | | 0.0003 | | | 1.75E-01 |
| rs11763020 | 7 | 1060288 | T | C | 0.0075 | 146.7557 | 0.5932 | | 0.0490 | | 2.49E-33 | -0.0002 | | 0.0002 | | | 2.06E-01 |
| rs11763793 | 7 | 1094342 | G | A | 0.0079 | 154.4576 | 0.5978 | | 0.0481 | | 5.76E-35 | -0.0003 | | 0.0002 | | | 1.34E-01 |
| rs11974176 | 7 | 1065569 | T | C | 0.0080 | 91.7664 | 0.3779 | | 0.0395 | | 1.47E-21 | -0.0002 | | 0.0001 | | | 2.77E-01 |
| rs12056053 | 7 | 1197632 | C | A | 0.0071 | 74.7265 | 0.3226 | | 0.0373 | | 7.11E-18 | -0.0001 | | 0.0001 | | | 5.50E-01 |
| rs12381396 | 7 | 1046777 | G | A | 0.0012 | 37.8026 | 0.4616 | | 0.0751 | | 8.41E-10 | -0.0001 | | 0.0002 | | | 6.96E-01 |
| rs12701834 | 7 | 1105426 | G | A | 0.0091 | 105.4188 | 0.4065 | | 0.0396 | | 1.69E-24 | 0.0000 | | 0.0001 | | | 8.92E-01 |
| rs12702016 | 7 | 1129392 | T | C | 0.0047 | 52.1727 | 0.2657 | | 0.0368 | | 5.82E-13 | -0.0001 | | 0.0001 | | | 4.74E-01 |
| rs12702029 | 7 | 1130584 | C | T | 0.0017 | 17.4889 | 0.1730 | | 0.0414 | | 2.94E-05 | 0.0001 | | 0.0001 | | | 3.34E-01 |
| rs13232402 | 7 | 1055598 | A | G | 0.0030 | 38.3482 | 0.2446 | | 0.0395 | | 6.37E-10 | 0.0000 | | 0.0001 | | | 8.70E-01 |
| rs17234336 | 7 | 1220005 | T | C | 0.0014 | 52.9026 | 0.8496 | | 0.1168 | | 4.03E-13 | -0.0001 | | 0.0003 | | | 7.46E-01 |
| rs1997243 | 7 | 1083777 | G | A | 0.0078 | 152.9682 | 0.5940 | | 0.0480 | | 1.19E-34 | -0.0003 | | 0.0002 | | | 1.66E-01 |
| rs2070118 | 7 | 1132505 | A | G | 0.0079 | 111.8326 | 0.4289 | | 0.0406 | | 7.12E-26 | -0.0003 | | 0.0002 | | | 4.86E-02 |
| rs3735686 | 7 | 1062527 | A | G | 0.0039 | 41.2351 | 0.2355 | | 0.0367 | | 1.47E-10 | -0.0001 | | 0.0001 | | | 5.43E-01 |
| rs3824080 | 7 | 939324 | T | C | 0.0006 | 16.5950 | 2.8142 | | 0.6908 | | 4.70E-05 | -0.0004 | | 0.0002 | | | 2.64E-02 |
| rs4722935 | 7 | 994621 | C | T | 0.0013 | 21.2815 | 0.6289 | | 0.1363 | | 4.06E-06 | 0.0000 | | 0.0002 | | | 8.12E-01 |
| rs4724104 | 7 | 1120918 | C | T | 0.0018 | 32.7551 | 0.5754 | | 0.1005 | | 1.10E-08 | -0.0001 | | 0.0002 | | | 5.55E-01 |
| rs4724463 | 7 | 1160190 | G | A | 0.0067 | 87.7125 | 0.3668 | | 0.0392 | | 1.10E-20 | -0.0003 | | 0.0002 | | | 9.80E-02 |
| rs6945202 | 7 | 1116558 | C | T | 0.0078 | 82.2461 | 0.3500 | | 0.0386 | | 1.67E-19 | 0.0000 | | 0.0001 | | | 8.27E-01 |
| rs6947257 | 7 | 1046906 | C | T | 0.0041 | 44.3119 | 0.2516 | | 0.0378 | | 3.09E-11 | 0.0000 | | 0.0001 | | | 8.31E-01 |
| rs6952546 | 7 | 988009 | A | G | 0.0066 | 136.1030 | 1.5070 | | 0.1292 | | 4.61E-31 | -0.0003 | | 0.0002 | | | 1.63E-01 |
| rs7784043 | 7 | 1210554 | G | T | 0.0062 | 65.5545 | 0.3002 | | 0.0371 | | 6.98E-16 | 0.0001 | | 0.0001 | | | 4.26E-01 |
| rs7784607 | 7 | 1178694 | G | A | 0.0057 | 62.7349 | 0.2999 | | 0.0379 | | 2.87E-15 | -0.0001 | | 0.0001 | | | 5.16E-01 |
| rs7787534 | 7 | 1225076 | C | A | 0.0018 | 22.4494 | 0.2961 | | 0.0625 | | 2.22E-06 | -0.0003 | | 0.0002 | | | 2.46E-02 |
| rs7807150 | 7 | 1020272 | A | G | 0.0022 | 51.7302 | 0.4001 | | 0.0556 | | 7.27E-13 | -0.0001 | | 0.0002 | | | 7.78E-01 |
| rs7809203 | 7 | 1087454 | C | T | 0.0058 | 60.9918 | 0.2802 | | 0.0359 | | 6.89E-15 | -0.0001 | | 0.0001 | | | 5.50E-01 |
| rs7811597 | 7 | 985996 | A | G | 0.0033 | 35.6503 | 0.7841 | | 0.1313 | | 2.52E-09 | -0.0001 | | 0.0001 | | | 3.77E-01 |
| rs9640009 | 7 | 1231861 | T | C | 0.0017 | 18.1666 | 0.1650 | | 0.0387 | | 2.06E-05 | -0.0003 | | 0.0001 | | | 5.35E-02 |
| rs9655470 | 7 | 1151713 | T | G | 0.0072 | 74.8350 | 0.3065 | | 0.0354 | | 6.73E-18 | 0.0000 | | 0.0001 | | | 7.39E-01 |

SNP, single nucleotide polymorphism; A1, effect allele; A2, other allele; se, standard error.

**Figure S1.** Scatter Plots of MR Analyses for miRNAs Causally Associated with CRS. (A-C) Scatter plots for miRNAs in the Ebi discovery cohort; (D-F) Scatter plots for miRNAs in the UKB replication cohort.
